# Dendrimer-Based Drug Delivery Systems for Brain Targeting

**DOI:** 10.3390/biom9120790

**Published:** 2019-11-27

**Authors:** Yuefei Zhu, Chunying Liu, Zhiqing Pang

**Affiliations:** 1 Key Laboratory of Smart Drug Delivery, School of Pharmacy, Fudan University, Ministry of Education, 826 Zhangheng Road, Shanghai 201203, China; yz3713@columbia.edu (Y.Z.); 19211030014@fudan.edu.cn (C.L.); 2 Department of Biomedical Engineering, Columbia University Medical Center, 3960 Broadway, New York, NY 10032, USA

**Keywords:** dendrimer, brain targeting, drug delivery, nanoparticles

## Abstract

Human neuroscience has made remarkable progress in understanding basic aspects of functional organization; it is a renowned fact that the blood–brain barrier (BBB) impedes the permeation and access of most drugs to central nervous system (CNS) and that many neurological diseases remain undertreated. Therefore, a number of nanocarriers have been designed over the past few decades to deliver drugs to the brain. Among these nanomaterials, dendrimers have procured an enormous attention from scholars because of their nanoscale uniform size, ease of multi-functionalization, and available internal cavities. As hyper-branched 3D macromolecules, dendrimers can be maneuvered to transport diverse therapeutic agents, incorporating small molecules, peptides, and genes; diminishing their cytotoxicity; and improving their efficacy. Herein, the present review will give exhaustive details of extensive researches in the field of dendrimer-based vehicles to deliver drugs through the BBB in a secure and effectual manner. It is also a souvenir in commemorating Donald A. Tomalia on his 80th birthday.

## 1. Introduction

Brain diseases are an ever-accelerated challenge in medical care since, with the aging of the world population, the number of patients with brain diseases will multiply, accompanying high social repercussion on account of grievous morbidity and mortality [1]. For instance, Alzheimer’s and Parkinson’s diseases are the two most common neurodegenerative diseases [2]. Moreover, glioma accounts for 80% of all malignant brain tumors and possesses characteristics of rapid onset and intense aggressiveness [3]. The concurrent therapy toolkit for cerebral tumors—surgery, radiation, and chemotherapy—falls woefully short for most people with the condition, for whom survival time is often measured in months [4]. Despite the possible therapeutic molecules derived from prolific scientific achievements [5], sufficient treatments remain an unmet medical demand because systemically administered agents are often inept by reason of a well-versed biological impediment: the blood–brain barrier (BBB) [1]. Amid multifarious organs of the human body, the brain is exceptionally adept at keeping out most therapeutic agents by means of this unique assembly of blood vessels which represents a multicellular interface that separates blood flow from brain parenchyma [1]. Although the BBB is crucial for maintaining brain health and this border is befitting for protecting the brain neurons against harmful and toxic agents that exist in blood, it also blocks the penetrance and access of many therapeutic agents [6]. In other words, it is also the main reason why treatments for cancer that work elsewhere in the body fail routinely when directed at the brain. Composed of brain capillary endothelial cells, pericytes, astrocytes, and neurons, the BBB is not a static wall; instead, it actively pumps selected molecules into or out of the brain. The passage of molecules to slip across the BBB relies primarily on their structure, surface properties, and chemical composition, allowing only low M_W_ (<400–500 Da) and lipophilic small molecules into the brain, thereby making traversing the BBB a particular challenge for the large, lipid-insoluble biological drugs [7]. Over 98% of small-molecule drugs and nearly 100% of large-molecule drugs such as recombinant proteins and monoclonal antibodies cannot enter the brain [8]. Currently, there are several feasible treatments for the central nervous system (CNS). Either the neuroactive agents can bypass the BBB after administration or they must be applied via invasive approaches referring to a high risk of rock-ribbed side effects [1]. Conceivable methods are, for instance, unfolding of the tight junctions via ultrasound [9] or osmotic disruption [10] and direct intracerebral infusing or implantation [11]. Since the BBB integrity is pivotal for the correct functioning of the CNS, in a small minority of cases, such as cerebral cancer or traumatic brain injury, the pathological mechanisms by themselves, influencing the BBB integrity, might endow drugs with the probability to reach the CNS in the progression of these diseases [12,13].

Since substantially every neuron possesses its own connection to a microvessel [14], the means to deliver drugs through the neurovascular unit (NVU) interface turn out to be one of the most promising tactics for efficient brain targeting [15]. In recent years, major focus of pharmaceutical, medicinal, and biophysical research has been navigated in exploring and developing novel and simple avenues to achieve enhanced efficacy of the administered agents via investigating various nanoparticle (NP) types, leading to an extensive comprehension of the mechanism of NP uptake in the brain [16]. Among these achievements, dendrimers exhibited great potential for a noninvasive treatment [1]. As an emerging class of synthetic and multifunctional polymers that possess an architectural structure resembling a tree or dendron, dendrimers and dendritic nanomaterials have garnered widespread concern mainly owing to their unique molecular architectures, multifunctional capabilities, and ease of surface modification with multiple functional agents (e.g., targeting ligands) [17]. Ever since Donald A. Tomalia published the synthesis and full characterization of a neoteric class of poly(amidoamine) (PAMAM) macromolecules and referred to these hyperbranched polymers as dendrimers in 1984, these nanoscopic compounds have been intensively studied for therapeutic use [18]. A wide variety of dendrimers with disparate structures and functions have been developed. Of particular note is that this significant contribution of Tomalia inaugurated a span-new field of research referring to nanotechnological approaches. In comparison with most polymers, many dendrimers are regarded as well-defined, discrete tunable macromolecules with high molecular uniformity and monodispersity. With copious internal cavities and surface functionalities, dendritic materials have been considered as promising vehicles for specific drug delivery [19]. Several cases of intrinsic dendrimeric therapeutic potential have been proposed such as antiprion [20,21], antitoxin [22], and anti-amyloidogenic effects [23]. Linking drugs and bioactive compounds or embedding them into a dendritic molecular frame can perfect many correlative biological features, such as bioavailability, solubility, and selectivity. In this regard, dendrimers stand for a perfect platform for brain drug delivery.

Many excellent reviews regarding the preclinical studies and potential applications of dendrimers are available [24,25,26,27]. Dendrimers are by themselves an intriguing class of nano-vectors for drug delivery toward brain diseases. In this review, we will briefly describe the physiochemical and biological properties of dendritic polymers and their unique synthetic strategies. In honor of the great contribution of Tomalia on his 80th birthday, this review will also retrospect structural and safety aspects of PAMAM and its derivatives. Moreover, emphasis has been given to brain drug delivery of dendrimer-based nanovehicles. Our staple objective here is to offer insight into the opportunities and challenges related to successful brain drug delivery in a format that is easily accessible to the diverse range of researchers. We will refer to technologies that are commercially available and highlight the momentous developmental milestones and the recent formulation advances achieved in dendrimer-based nanovehicles. Ultimately, we will delineate the challenges that dendrimers have faced in their clinical translation and discuss how those challenges could be potentially addressed.

## 2. Physiochemical and Biological Properties of Dendrimers

**Figure 1:**
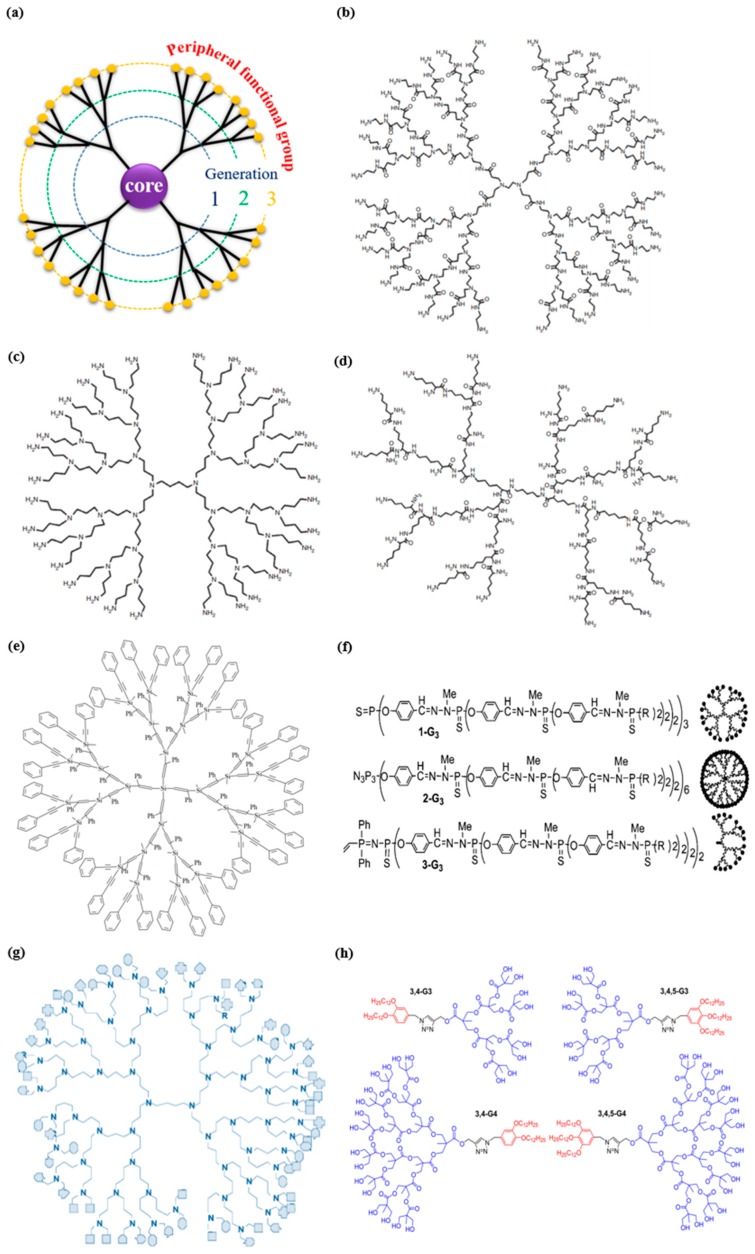
Commonly used dendritic macromolecules in drug delivery: (**a**) A schematic diagram revealing the three components of a dendrimer molecule and chemical structures of (**b**) polyamidoamine (PAMAM), (**c**) polypropyleneimine (PPI), (**d**) poly-l-lysine (PLL) dendrimers, and (**e**) carbosilane dendrimers; (**f**) examples of cores of phosphorus (PPH) dendrimers, (**g**) glycodendrimers, and (**h**) Janus dendrimers. Figure 1**b**–**h** is reproduced with permission from References [35,36,37,38,39].

Dendrimers are nanosized macromolecules characterized by hyper-branched globular structure and widely used for drug delivery. Compared with traditional polymer nanovehicles, dendrimers are of monodispersity and have well-defined chemical structures. Additionally, the specific structure of dendrimers endows them flexibility to load therapeutic drugs by either covalent conjugation or electrostatic adsorption [28]. Dendrimers are semblable in size to a crowd of biological structures. For instance, generation 5 (G5) polyamidoamine (PAMAM) dendrimers are approximately the same size and shape as hemoglobin (5.5 nm in diameter) [29]. Generally, dendrimers consist of a centric core (single atom or group of atoms), repeating building units attached to the core and known as generations, and functional groups on the surface (Figure 1a). Therefore, physiochemical and biological characteristics of dendrimers are determined by virtue of three structural components of dendrimers: core, building blocks, and functional groups. There are two common synthetic methods to prepare a dendrimer: divergent and convergent. In the divergent approach, dendrimers are constructed from the core to the shell, generation by generation, while in the convergent approach, compounds are constructed from the periphery to the core.

The surface groups may have positive, negative, and neutral charges, which is important in probing suitable dendrimers as desired drug delivery carriers [30]. Dendrimers terminated with positively charged functional groups usually cause destabilization of anionic cell membrane and even cell lysis, which leads to low biocompatibility, whereas neutral or anionic charged dendrimers exhibit comparatively less hemolysis [31]. To overcome this issue, surface functionalization such as PEGylation may bring about alterations in zeta-potential, blood retention, and even distribution in vivo. Gene therapy is a burgeoning promising approach for a great deal of diseases; nevertheless, it still encounters some obstacles in the inability of the free gene molecules to access the target cells in vivo [32]. The cationic dendrimers electrostatically attract the negatively charged polynucleotides to form stable dendriplexes. The dendriplexes are released through a “proton sponge” effect in endosomes after cellular uptake, facilitating gene expression in cells [33,34]. As gene delivery carriers, dendrimers can protect gene molecules from biodegradation, can facilitate nucleic acids to penetrate into cells, and can maintain biological activity of gene molecules.

## 3. Dendrimers and Dendrimer Derivatives

### 3.1. PAMAM Dendrimers

The term “polyamidoamine dendrimers” (PAMAM) is classically intended to refer to dendrimers comprised of (i) a core (earliest core: an ethylenediamine core), (ii) branches made up of amide groups emanating from a branching point that forms the walls of cavities, and (iii) amine functional groups on the periphery (Figure 1b). Concurrently, there is a large selection of PAMAM dendrimers with disparate surface groups [40]. PAMAM dendrimers, one of the most fully studied dendrimers, possess internal cavities and peripheral functional groups, which can be further modified to encapsulate agents or other cargos for biomedical applications. As the dendrimer inventor Donald Tomalia has indicated, dendrimers could serve as nano-agents against tumors, bacteria, and viruses [41]. However, one concern over the use of PAMAM, especially those terminated with positively charged groups, is their cytotoxicity, which is concentration and generation dependent in vitro. G5 or lower generation PAMAM dendrimers are considered nontoxic [42]. To overcome this limit, surface modification with PEG has been made. Compared with other dendrimers, PAMAM has the widest applications in the field of drug delivery, antimicrobial, antiviral, and antioxidant agents for diagnosis and drug targeting.

### 3.2. PPI Dendrimers

Polypropyleneimine (PPI) dendrimer is the oldest known dendrimer commercially used for drug delivery. It consists of a diaminobutane core and propyleneimine repeat units (Figure 1c). Because diaminobutane (DAB) constitutes a core of PPI, DAB-dendrimer is the second most popular abbreviation of polypropyleneimine [40]. The amino terminal groups of PPI provide adequate solubility in water. This enables PPI dendrimers to raise the aqueous solubility of hydrophobic agents entrapped in the hydrophobic interior cavities of PPI. However, the positively charged surface of PPI often destabilizes cell membranes and causes cell lysis. Another problem is the lower drug loading capacity of PPI compared with PAMAM [43]. The PPI/drug complex is less stable than PPI. Surface group modifications such as PEGylation and acetylation have been chosen. Acetylation is preferred for its high efficiency and high penetration ability. Moreover, the steric hindrance of PEG chain can influence the interaction of surface functional groups with drug molecules [44].

### 3.3. PLL Dendrimers

Poly-l-lysine (PLL) dendrimers (also called dendri-grafted poly-l-lysine or DGL) are intended to refer to those dendrimers comprised of lysine residues (Figure 1d). These dendrimers offer the advantages of improved biocompatibility, low cytotoxicity, facile enzymatic degradation, and subsequent excretion of low molecular weight products [45]. Moreover, the capacity to introduce stimuli responsiveness when required through inclusion of specific amino acids sequences of PLL dendrimers is very appealing [46]. PLL dendrimers and their derivatives have been extensively used in gene delivery. The higher generation of PLL exhibits better gene transfection, while the traditional linear poly(lysine) shows lower gene transfection efficiency and elevated cytotoxicity compared with PLL dendrimers.

### 3.4. Carbosilane Dendrimers

With the extensive application of silicon chemistry, a series of carbosilane dendritic macromolecules which possess hydrophobic scaffolds and remarkable thermal stability have also been introduced [47]. The preponderance of utilizing silicon chemistry to synthesize dendrimers rests with the fact that nucleophilic molecules can readily access electrophilic silicon (Si^+^) [48]. Notably, the superiority of carbosilane dendrimers is associated with the low polarity as well as the high energy of the C–Si bond, endowing it with high hydrophobicity [49]. Although carbosilane dendrimers possess such a hydrophobic internal skeleton, they can still be converted into hydrophilic compounds via surface functionalization with polar moieties. Reactive groups, like Si–H, Si–Cl, Si–CH=CH_2_, and Si–CH_2_CH=CH_2_, can help introduce many other intriguing inorganic, organic, and organometallic substituents, leading to the increasing application in pharmaceutic fields [40].

### 3.5. PPH Dendrimers

Among the family of cationic dendrimers, phosphorus (PPH) dendrimers, which are synthesized especially for the drug delivery systems (DDSs), are also of particular interest. PPH dendrimers, that is, dendrimers owning phosphorus atoms at each branching point, hold a special place on account of their relatively straightforward fabrication and the rich chemistry diversity, in most cases owing to the presence of reactive end groups (aldehydes or P(S)Cl_2_ groups) [37]. Such dendrimers can possess a hydrophilic surface and a hydrophobic backbone, which permits them to efficiently interplay with cell membranes and to be internalized into cells [50,51]. Recent researches have revealed that PPH dendrimers exhibit great potentials in biomedical applications. It has been verified that such dendritic materials can influence amyloid peptide and Tau protein aggregation in neurodegenerative diseases [52]. Moreover, the potential of PPH dendrimers in delivering anticancer siRNAs to target cells [53] and gene therapy against HIV infection [54] have also been reported.

### 3.6. Janus Dendrimers

Recently, “Janus” dendrimers, also called bow-tie dendrimers, diblock dendrimers, co-dendrimers, and “surface-block” dendrimers and characterized by two dendrimeric wedges of different terminal groups, are a relatively new class of amphiphilic dendrimers for pharmaceutical applications. The name “Janus” refers to the ancient God of gates and doors, generally depicted as having a two-faced head, facing opposite directions [55]. The broken symmetry of Janus dendrimers provides the opportunity to form complex self-assembled materials and presents new sets of features that are presently inconceivable for homogeneous or symmetrical dendrimers. Therefore, Janus dendrimers, of which the properties are directly linked to the two different peripheral surface groups, are very desirable. These Janus dendrimers gain interest particularly in self-assembling and medical applications due to their biofunctional character and thermal properties [56].

### 3.7. Linear-Dendritic Block Copolymers

With the development of dendrimers, there exists a novel amphiphilic block copolymer, linear-dendritic block copolymers (LDBC). They consist of linear chains covalently attached to the dendrimer. Most of LDBCs are involved in the conjugation of hydrophobic dendrons to hydrophilic linear chains [57]. The amphiphilic copolymers could self-assemble in aqueous solution to form stable core-shell structure, which exhibits remarkable features distinguished from conventional micelles (e.g., lower critical micelle concentration (CMC) and prolonged stability in the blood) [58]. In terms of architecture, copolymers can be split into six prime groups: AB diblock linear-dendritic copolymers which contain the linear A block and dendritic B block, ABA triblock linear-dendritic copolymers which employ B as the linear block and A as the dendritic block, linear-hyperbranched polymers, side chain functional or dendronized linear-dendritic copolymers, multi-arm star copolymers, and linear-dendrimer-grafts polymers [58].

### 3.8. Other Types of Dendrimers

Aside from the aforementioned LDBC dendrimers, which belong to the hybrid dendrimer family, many other kinds of dendrimers with well-defined nanoscopic size and ample number of functional terminal groups have become available. These dendrimers include core shell (tecto) dendrimers (a polymeric architecture with highly ordered structure achieved via controlled covalent attachment of dendrimer building blocks) [59], peptide dendrimers [60], glycodendrimers [61], PAMAM-organosilicon (PAMAMOS) dendrimers (consists of hydrophilic and nucleophilic PAMAM interiors and hydrophobic organosilicon (OS) exteriors) [62], and so on.

## 4. Strategies of Dendrimer-Based Nanocarriers for Brain Targeting

Extensive studies have shown that nanomaterials have been successfully employed for the treatments of brain diseases. Brain drug delivery can be sorted into two staple domains: bypassing the BBB and traversing the BBB. Based on the various structures and properties of dendritic polymers, drug molecules can either be physically encased into the internal cavities of dendritic molecules or chemically conjugated to the terminal functional groups [63]. Tailoring of the peripheral functionalities of dendrimers can be regarded as a sparing method to impart new features. Surface-engineered dendrimers will obtain enhanced biocompatibility, drug-release kinetics, and aptitude to target the BBB or brain tumors and to expedite transportation of bioactive agents across the BBB.

Section 4.1 and Section 4.2 will shed light on a compendious overview of the multifarious tactics that can be applied to deliver drugs to the CNS without the need to construct the BBB-crossover functions. Additionally, there are some endogenous pathways that can be utilized to facilitate drug passage through the BBB, and these are discussed in the ensuing Section 4.3. This section will provide direct insight into the disparate pathways at length and goes in quest of highlighting specific drug modification and of targeting tactics that can leverage these multifarious pathways.

### 4.1. Bypassing the BBB with Invasive Approaches

As for the current scientific research, invasive approaches permit the direct transfer of the drug into the cerebral lesions, incorporating intracerebroventricular (ICV) [64], intracerebral/intraparenchymal administration [65], convection-enhanced delivery (CED) [66,67], and intrathecal [68] and intratympanic administration [69].

ICV administration is a well-established and well-tolerated method for extended brain drug delivery [70], through which the delivered drug is introduced from an implantable reservoir or through an outlet catheter drawn from a pump [71]. It is evident that ICV administration may diminish systemic toxicity and may shun drug metabolism in the blood serum. However, such a method may also induce some significant drawbacks and risks. Any drug injected by means of ICV administration can only penetrate the brain parenchyma via a sluggish diffusion process. In addition, other related risks that need to be considered incorporate infections and incremental intracranial pressure owing to fluid injection [65].

CED, as a local delivery tactic for the CNS, was initially termed by Bobo et al. in 1994 [66]. CED provides several preponderances over diffusion-mediated delivery, such as enhanced intratumoural spatial distribution, less toxic doses, independence of the molecular weight, or diffusivity of drugs [72]. For instance, as early as 2002, CED was explored to enhance the intracerebral and intratumoural uptake of a heavily boronated macromolecule dendrimer conjugated to epidermal growth factor (EGF) for neutron capture therapy in rats bearing a syngeneic epidermal growth factor receptor (EGFR)-positive glioma [73]. The outcomes indicated that CED was more effective than intratumoural injection to deliver boronated EGF to EGFR(+) gliomas for boron neutron capture therapy. Overtly, in comparison with other neurosurgical measures, the locoregional distribution of CED rendered it a more promising tactic for clinical application [74]. Although CED holds great potential in brain drug delivery, limited success has been achieved in clinical trials. Several concerns must be considered for the preclinical and clinical development. First, invasive procedures may appear with the risk of infection. Second, high pressures in connection with convective flow can induce the undesired diversion of fluid to more sensitive and less flow-resistant regions (e.g., the subarachnoid space) [75]. Third, technical shortcomings associated with CED need to be addressed [72] and comprehensive studies are necessary to further validate the safety and effectiveness of CED in clinical practice.

Similar to ICV, intracerebral or intraparenchymal administration involves the direct delivery of drugs to the brain parenchyma by implantation or injection [65]. Nevertheless, compared with ICV, this process may not be an ideal method because of its limitation that drug molecules will rarely penetrate into ambient tissues. Similarly, although intrathecal administration has been applied to treat a broad range of brain disorders for many decades, it still suffers several disadvantages in clinical applications. It is arduous to forecast the percentage of an intrathecal dose reaching the brain, and treatment is dependent on reliable catheter placement and stability [76,77].

Although the aforementioned invasive methods could deliver drugs into the brain, these approaches are gradually falling out of favor on account of risks, such as void of targeting and traumatic and some other surgical complications. Consequently, more and more dendrimer-based nanoparticle delivery systems turn to transport to the brain via noninvasive approaches [78].

### 4.2. Bypassing the BBB with Noninvasive Approaches

In contrast to most other means delineated in the foregoing section, intranasal delivery is a noninvasive way that does not need an injection [79]. Drugs via nasal administration can be assimilated into the systemic circulation without enzyme degradation at a great lick and hepatic first-pass effect that is usually associated with oral administration, thereby enhancing the bioavailability and bringing about fleet onset of the pharmacological effect [80,81]. More importantly, nasal administration can allow direct delivery of many drugs into the CNS through the direct anatomical connection between the nasal cavity and the brain without crossing the BBB [82,83,84,85,86,87]. It is hypothesized that drugs deposited at the nasal mucosa mainly travel to the brain via the olfactory neurons and via the trigeminal nerves [85,88]. The nose-to-brain delivery has been displayed in both preclinical and clinical studies with a variety of formulations such as nasal sprays, powders, gels, etc. [89].

Nanoparticle-based drug delivery systems can facilitate nose-to-brain delivery of drugs as nanoparticles could shield drugs from degradation, could enhance retention in the nasal cavity, and could prevent drug efflux back into the nasal cavity. In this regard, dendrimers may possess great potential to deliver drugs to the brain by nasal administration [90]. For instance, Kim et al. explored the therapeutic effect of intranasal small siRNA delivery in the postischemic rat brain by leveraging high mobility group box-1 (HMGB1) as the target. Specifically, siRNA was complexed with a biodegradable PAMAM dendrimer as a gene vector. It was shown that fluorescent-labeled siRNA appeared in the cytoplasm and processes of neurons and of glial cells in many brain regions, incorporating the amygdala, cerebral cortex, and striatum at 1 h after nasal administration. More significantly, nasal delivery of HMGB1 siRNA markedly decreased the cerebral infarct volume in rats after cerebral ischemia by a maximum of approximately 43% and recovered from neurological and behavioral deficits, showing great potential of nasal gene delivery to the brain with dendrimers [91].

Nose-to-brain delivery has been testified to be a handy, noninvasive, and cost-efficient choice for direct transport of drugs to the brain. However, nose-to-brain delivery is most effective for highly potent drugs that are effective in the brain at nanomolar concentrations or at even lower concentrations. In addition, there are still some challenges to overcome for nose-to-brain delivery such as obstruction of the nasal epithelial barrier, rapid clearance from nasal cavity, limited penetration of drugs from nerve entry points [68,92], and nasal absorption irregularities during disease states.

### 4.3. Traversing the BBB with Noninvasive Approaches

**Figure 2:**
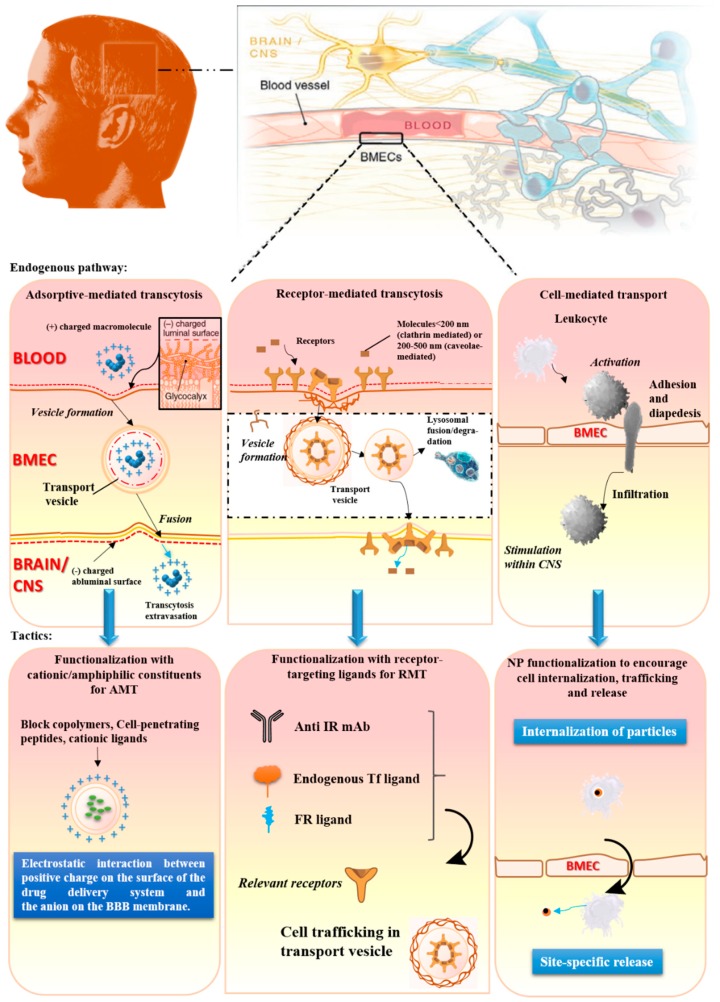
Noninvasive central nervous system (CNS) drug-delivery tactics that leverage endogenous pathways to traverse the blood–brain barrier (BBB): The major pathways incorporate adsorptive-mediated transcytosis (AMT), receptor-mediated transcytosis (RMT), and cell-mediated transport are presented. Modified and reproduced with permission from Reference [71].

In the context of dendrimer-based DDSs, the noninvasive approaches through which molecules can traverse the BBB and enter the CNS mainly incorporate carrier-mediated transport (CMT), adsorptive-mediated transcytosis (AMT), receptor-mediated transcytosis (RMT), paracellular transport, passive transcellular diffusion, and cell-mediated transport [71,93]. The schematic diagram of three major transport systems is displayed in Figure 2.

Of these, the CMT mechanism, involving the binding of an endogenous solute to a protein carrier or transporter on the BBB luminal side, can mediate the entry of major nutrients like hexose, monocarboxylic acid, glucose, vitamin, amino acid, and nucleotides into the brain [78]. Therefore, small molecules or nanoparticles conjugated to these nutrients may be delivered across the BBB via CMT. For instance, a research built on the hydroxyl-terminated G4 PAMAM dendrimers (D4-OH) for penetrating the impaired BBB and for targeting activated glia certified that the CMT strategy was a hopeful approach [94]. Sharma et al. conjugated mannose to the surface of versatile D4-OH with highly efficient click chemistry to explore whether coupling target ligands could promote brain uptake. The outcomes of in vitro experiments indicated that mannose modification significantly changed the internalization mechanism of dendrimers, making them more likely to undergo carrier-mediated mannose endocytosis than nonspecific liquid phase endocytosis [94]. Transporters on the BBB generally involved in brain drug delivery via CMT include glucose transporter 1 [95], large neutral amino acid transporter 1 [96], cationic amino acid transporter 1 [97], nucleobase transporter [98], monocarboxylic acid transporter 1 [99], choline transporter [99], etc.

AMT (Figure 2), a vesicle transport system, involves the endocytotic internalization of positive macromolecules or nanoparticles, followed by their subsequent passage through the BBB [100]. In theory, AMT can be realized via establishing cationic charge in the drug or nanoparticles or through conjugating the drug or nanoparticles with a positively charged moiety [101]. The disadvantages associated with AMT involve void of selectivity, meaning that adsorption may occur not only in the BBB but also in the blood vessels of other organs, and the possibility of increasing the BBB permeability, which may be ascribed to the toxic effects of positively charged compounds (e.g., large doses of cell penetrating peptides).

Compared to AMT, RMT (Figure 2) is a pathway that features higher affinity and higher transcytotic potential. It is another promising approach for brain drug delivery of macromolecule drugs. Endocytosis occurs, and membrane invagination brings about the formation of vesicles that contain receptor-ligand conjugates [78], followed by subsequent ligand passage through the BBB. For the past few years, the dendrimer-based DDSs by RMT is in vogue for overcoming the BBB issue. Receptors on the BBB generally involved in brain drug delivery via RMT include insulin receptor [97], transferrin receptor [102,103,104], nicotinic acetylcholine receptor [105], insulin-like growth factor receptor [102], diphtheria toxin receptor [102], neonatal Fc receptor [98], scavenger receptor [102], receptor of advanced glycosylation endproducts (RAGE) [106], apolipoprotein E receptor 2 [107], melanotransferrin receptor [108], low-density lipoprotein receptor-related protein-1 (LRP1) [109,110] [111], etc. Several present investigations seek to develop a variety of ligand-anchored dendrimers and to compare their brain-targeting potential on a single platform. Sialic acid, concanavalin A, and glucosamine-anchored PPI dendritic nanoconjugates were assessed for the brain delivery of an antitumor agent, paclitaxel (PTX) [112]. The order of brain-targeting potential of disparate ligands was found to be concanavalin A < glucosamine < sialic acid. Therefore, sialic acid can be utilized as a potential ligand, appending PPI to enhance anticancer drug delivery to the brain, thus achieving higher therapeutic effects [112]. Since transferrin carrying Fe^3+^ can effectively reach the brain via transferrin receptor-mediated transcytosis [113], a transferrin (Tf)-bearing G3 PPI dendrimer was prepared for brain gene delivery. It is noteworthy that the nanocarrier system can increase gene uptake and gene expression in tumor cells overexpressing transferrin receptors in contrast with the non-targeted delivery system [114]. Similarly, lactoferrin, a cationic iron-binding glycoprotein which belongs to Tf family, has also been exploited to form a lactoferrin-bearing G3 PPI dendrimer [115]. It was shown that the conjugation of lactoferrin to the dendrimer brought about an enhanced pDNA uptake by 2.1-fold in bEnd.3 murine brain capillary endothelial cells (BCECs) opposed to the unmodified one in vitro [115]. Therefore, it is remarkable that dendrimers can be adept at targeting the brain via RMT, and with the advent of multifunctional dendrimers along with the proof that targeting can alter biodistribution and dendrimer-cell interactions, the potential for dendrimer application in theranostics for brain diseases is abidingly expanding.

## 5. Design and Application of Dendrimer-Based DDSs For Brain Targeting

This section will consider the design and application of dendrimer-based brain delivery systems and the in-depth research with respect to the modifications made on drugs to prompt their brain uptake. Conceivably, many brain-targeting tactics exploiting free drugs can be applied equally to nanoparticles, and hence, some of the tactics probed and discussed in this section may overlap with some concepts in the aforementioned portion (incorporating several noninvasive methods). For the sake of succinctness and brevity along with preventing superfluous repetition, the tactics that incur superposition will only be discussed at length once in the section wherein they are more apt to be exploited. The entire discovery and developed dendrimer-based DDSs are listed in Table 1.

### 5.1. Brain Delivery of Small-Molecule Drugs with Dendrimers

For small molecules, they can be encapsulated or covalently attached to the surface of dendrimers [141]. The open structure of dendrimers provides a possibility to entrap drug molecules within the cavities of a dendrimer. Largely, lower generation dendrimers tend to possess a patulous and amorphous structure, but in the wake of generation increasing, the structure becomes more compact and globular. The nature of encapsulation includes electrostatic interaction, simple physical entrapment, and hydrophobic and hydrogen bond interactions [141]. These interactions offer the potential of dendrimers to incorporate those erratic or insoluble drugs to enhance drug aqueous solubility and bioavailability and to control drug release. The number of drug molecules encapsulated within dendrimers relies on the structure of a dendrimer to a certain extent, while the surface loading capacity may markedly increase because of the formation of a composite with numerous functional groups. In general, each additional generation of dendrimers doubles the number of end groups available for surface interactions. However, not all end groups are accessible for interactions because of the steric hindrance or backfolding of chains into the dendritic architecture. The presence of ionizable groups on the periphery of dendrimers offers an opportunity for electrostatic attachment, which causes a considerable increase of drug solubility. The electrostatic interaction is often influenced by the surface charge density of dendrimers and ionic strength. Small-molecule drugs are often attached to dendrimers through hydrolysable or biodegradable linkages, which provide the potential of stepped-up control over drug release. The conjugation is often formed through a chemical spacer like polyethyleneglycol (PEG) and p-amino benzoic acid [142] via amide or ester linkages. It was found that the amide bonding is of better stability whereas ester bonding provides a way of controlling drug release through hydrolysis [42]. The hydrolysis of the conjugates leads to a slow release of the drug, thereby decreasing the toxic side effects of potent drugs. However, there is a problem of low water solubility of complexes arising from conjugating many drug molecules. This issue can be addressed by means of the surface attachment of short PEG chains.

As for brain tumors, most brain tumors are invasive and exhibit relatively weak, enhanced permeability and retention (EPR) effects in stark contrast with other peripheral solid tumors. Specifically, the vascular permeability of intracranial solid tumors is much lower than that of peripheral solid tumors, which may be due to the relatively few intracranial cavitation and vesicle-related organelles across the endothelial space [143]. It is estimated that the microvascular aperture of the U87 glioma ranges from 7 to 100 nm, which is significantly smaller than that of peripheral tumors (380–780 nm) [144]. Moreover, glycocalyx coating on the luminal surface of microvascular endothelial cells in brain tumors makes the true microvascular physiologic aperture smaller than its anatomic aperture [145]. Therefore, small-sized dendrimers may penetrate deeper and may obtain a more extensive tissue distribution in brain tumors than conventionally sized (approximately 100 nm) nanoparticles. Sarin et al. reported gadolinium (Gd)-diethyltriaminepentaacetic acid (DTPA)-chelated G5 PAMAM dendrimer conjugated with doxorubicin (DOX) by pH-sensitive covalent linkages (Gd-D5-DOX) as theranostics for brain tumors. It was demonstrated that Gd-D5-DOX ranging from 7 to 10 nm could deliver therapeutic concentrations of DOX across the BBB into individual brain tumor cells. The outcomes denoted that a single dose of Gd-G5-DOX was significantly more effective than free DOX with an equivalent dose in restraining the growth of RG-2 glioma [146]. To enhance nanoparticle retention in brain tumors, Zhao et al. conjugated PAMAM dendrimers with fibrin-binding peptide CREKA to construct a small nanoparticle DDS targeting the extracellular fibrin in brain tumors. Distinctly, in comparison with unmodified PAMAM, CREKA-modified PAMAM attained higher accumulation and deeper penetration in glioblastoma multiforme (GBM) tissue [147], proving to be a hopeful tactic for brain tumor treatment. Moreover, some brain tumor capillaries overexpress several receptors, guiding ligand-anchored dendrimer-based DDSs and promoting the drug delivery to the brain tumor tissues [148]. It is reported that efficient active targeting drug delivery may rely on covalent attachment of particular ligands to drug vehicles that can be identified by antigens, receptors, or other molecules overexpressed on the target sites. In this regard, dendritic macromolecules possess distinct composition, good physical and chemical properties, and adjustable surface functions, which are conducive to active targeting drug delivery and thus become a successful tool for the therapy of brain diseases. It has been reported that polyether-copolyester (PEPE) dendrimers possess low toxicity, high permeability across the BBB, as well as long circulation half-life [149]. They can be internalized efficiently into brain vascular endothelial cells with several pathways, among which clathrin and caveolin-mediated endocytosis turn out to be the major contributors. Another intriguing study is associated with the RGD-modified PEGylated PAMAM dendrimer with DOX conjugated to PAMAM via the acid-sensitive cis-aconityl linkage, which could fortify tumor targeting through binding with the overexpressed integrin receptors on tumor cells and controlled DOX release in acidulous lysosomes [150]. The nanoconjugate manifested significantly higher accumulation in the brain tumor over the normal brain tissue in a glioma model.

As mentioned above, the most renowned hurdle lurking the dispiriting glioma treatment is the presence of the BBB, which prevents drug delivery to cancer cells invaded in the normal brain tissue where the BBB is relatively intact. Thus, in recent years, dual-targeting dendrimer nanoparticles have fascinated a lot of interest because these nanomedicines endowed with versatile functions could transport across the BBB and could reach cancer cells to improve the efficacy of brain tumor-targeted delivery [151]. For instance, a pH-sensitive dual-targeting drug nanoparticle was developed based upon G4 PAMAM dendrimers [123]. In this work, transferrin (Tf) and tamoxifen (TAM) were selected as the targeting ligands for reinforcing the BBB transporting capability and for congregating DOX into glioma cells, respectively. Such dual-targeting nanoparticles could effectively inhibit the growth of C6 glioma cells but could greatly reduce the cytotoxicity of DOX to normal cells [123]. To simplify the dual-targeting strategy, ligands binding to the common receptors expressed both on the BBB and the tumors (i.e., transferrin receptor [122] and LRP1 [124]) are conjugated to dendrimers for dual targeting drug delivery to cerebral tumors.

**Figure 3:**
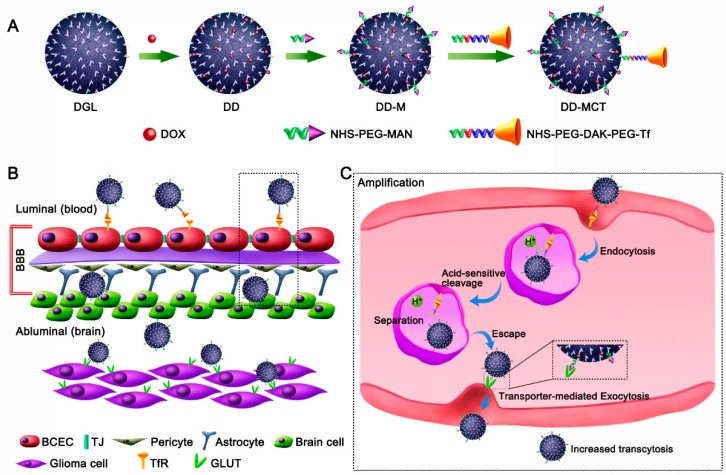
A dual targeting drug delivery system (DDS) for enhanced transport across the BBB and glioma targeting: (**A**) Diagram depicting the fabrication of acid-responsive programmed targeted DDS of DD-MCT. (**B**) Schematic elucidation of DD-MCT programmed targeting the transferrin receptor (TfR) on BBB and then targeting glucose transporter (GLUT) on glioma cells after transcytosis across BBB. (**C**) Schematic depiction of the procedure of the acid-responsive cleavage of transferrin (Tf) from DD-MCT in endothelial cells along with the transporter-mediated exocytosis of detached DD-M for enhanced transcytosis. DD-MCT, transferrin- and P-aminophenyl-α-d-mannopyranoside-decorated doxorubicin-loaded poly-l-lysine dendrimers); DD-M, P-aminophenyl-α-d-mannopyranoside-decorated doxorubicin-loaded poly-l-lysine dendrimers. Reproduced with permission from Reference [136].

RMT mechanism is normally utilized for nanoparticle DDSs to transport across the BBB. Notwithstanding, the transcytosis of ligand-modified NPs by RMT are apt to be trapped in brain capillary endothelial cells (BCECs) because of the high binding affinity of ligands with receptors. To perfect the BBB-traversing ability of nanotherapeutics, Ruan et al. designed a dual targeting DDS for programmed glioma targeting delivery, where P-aminophenyl-α-d-mannopyranoside (MAN) decorated doxorubicin-loaded DGL (DD-M) was covered with an acid-cleavable transferrin (Tf) coating (DD-MCT) (Figure 3) [136]. DD-MCT could specifically bind to Tf receptor (TfR) on the luminal side of the BBB endothelium, could cleave Tf after endocytosis, and could detach DD-M from the Tf–TfR complex in endo/lysosomes. DD-M could be inclined to get away from endo/lysosomes, could be exocytosized into brain parenchyma by the mediation of glucose transporter (GLUT) on the abluminal endothelial membrane, and could further be internalized into glioma cells by GLUT-mediated endocytosis. Significantly, DD-MCT transport into brain tumors was greatly enhanced, resulting in improved anti-glioma therapeutic outcome [136]. Considering smaller nanoparticles penetrate the BBB faster than bigger nanoparticles and most dendrimers have a uniform small size [152,153], this strategy paves a new way to enhance nanoparticles across the BBB for brain drug delivery.

Apart from the probing to the cerebral tumor, dendrimer-based DDSs also center on other cerebral diseases such as the Alzheimer’s disease [154], stroke, and cerebral palsy [155]. It has been shown that both dendrimer physicochemical properties and disease pathophysiology determines brain uptake, diffusion, and particular cellular uptake of dendrimers. It is reported that PAMAM sized less than 11 nm is optional in traversing the impaired BBB in an ischemic stroke model [156]. Neutral dendrimers could efficiently move in the brain parenchyma and could promptly be internalized in activated glial cells of the impaired site in a rabbit model of cerebral palsy [157,158]. Hydroxyl-modified G4 PAMAM can cross the damaged BBB and accumulated in the disease site of cerebral palsy, ischemic stroke, neonatal stroke, hypothermic circulatory arrest induced brain injury, and perinatal hypoxic-ischemic encephalopathy [155,159,160,161]. More importantly, dendrimer accumulation in the disease site is also dependent on the level of BBB damage, glial activation, and severity of brain disease [157]. The collapse of tight junction proteins ZO, occludin, and claudin-5 in the BBB may contribute to the transport of dendrimers into the brain parenchyma at the disease site [157].

Loading drugs to dendrimers would exert profound effects after they enter the diseased site or even specific cells that mediate neuroinflammation (i.e., microglia and astrocytes). For instance, researchers developed a dendrimer-*N*-acetyl-l-cysteine nanoconjugate (D-NAC) for cerebral palsy therapy [155]. In a cerebral palsy rabbit model, neuroinflammation in human brain and motor deficits in children were replicated. D-NAC treatment inhibited the neuroinflammation and led to a conspicuous enhancement in motor function in the cerebral palsy rabbit, thereby suggesting the opportunity to treat human cerebral palsy after birth [155]. The outcomes suggested that intravenous administration of such conjugates manifested colossal promise in the targeted therapy of neuroinflammation, especially in various preclinical models [162]. In addition, Venkata K’s group reported that D-NAC was stable for 6 h in all five simulated gastrointestinal fluids with no signs of chemical degradation [162]. These outcomes evince that an oral pediatric formulation containing D-NAC and glycerol monocaprylate may be an effective alternative for the neuroinflammation therapy.

### 5.2. Brain Gene Delivery with Dendrimers

Gene therapy is hopeful and encouraging for the remedy of neurological disorders or CNS injury [163]. However, gene therapy of cerebral diseases is confined owing to the void of a secure and efficient gene delivery system that can penetrate the BBB, thereby reaching the brain cells after intravenous administration. To elude the hurdles of translating virus gene transfection into clinical treatment, dendritic biomaterials appear to be particularly promising because of attractive attributes of dendrimers like the spherical architecture, malleable molecular size, and modifiable cationic groups. Furthermore, dendrimers have well-defined chemical structures and high density of cationic charges displaying electrostatic interplays with nucleic acids such as DNA, siRNA, and miRNA, formulating dendriplexes that can shelter the nucleic acids from degradation and can enhance gene transfection in cells [164]. Over the last few years, the performance of dendrimer-based gene delivery systems has gradually undergone optimization to meet the demand for lower cytotoxicity and higher efficacy. For instance, owing to the fact that the angiopep-2 peptide targeting LRP1 on the BBB exhibited high transcytosis capacity and parenchymal accumulation, Ke et al. modified PAMAM with angiopep-2 via bifunctional PEG (PAMAM–PEG–Angiopep) for efficient brain gene delivery [125]. Positive PAMAM–PEG–Angiopep was complexed with negative DNA, generating PAMAM–PEG–Angiopep/DNA NPs. The angiopep-2-modified NPs were noted to be internalized into BCECs by virtue of a clathrin- and caveolae-mediated energy-dependent endocytosis, also partly through macropinocytosis. Further results showed that such nanoparticles possessed higher efficiency in traversing the BBB in vitro and accumulated more in the brain in vivo compared with unmodified nanoparticles [125]. For this purpose, the angiopep-modified nanoparticles may be a prospective candidate in gene expression in the brain. Similarly, Liu et al. established an efficient brain-targeting gene DDS by modifying PAMAM with RVG29 peptide, a 29-amino-acid peptide stemmed from the rabies virus glycoprotein (RVG29) targeting the nicotinic acetylcholine receptor on the BBB [126]. RVG29 was modified on PAMAM via bifunctional PEG and then complexed with DNA, popping up the composite nanoparticles (PAMAM–PEG–RVG29/DNA NPs). The in vivo imaging indicated that PAMAM–PEG–RVG29/DNA NPs were more readily accumulated in the brain. Furthermore, the reporter gene expression of the PAMAM–PEG–RVG29/DNA nanoparticles was spotted in the brain and significantly higher than unmodified nanoparticles. Although the exact mechanism of this efficient gene delivery system had not been wholly interpreted, such a nano-vector still cradles prodigious promise as the nonviral vehicle for efficient, noninvasive, and brain gene delivery [126].

Another promising type of dendrimers to delivery siRNA across BBB is the amino-functional polyester dendrimers, which are known for their biocompatibility and the biodegradation of their internal esters [165]. Patrik et al. explored these dendrimers based upon 2,2-bis(methylol)propionic acid (bis-MPA) as nonviral vectors for siRNA delivery [140]. In this research, amino-functional bis-MPA dendrimers showed successful gene transfection in both rat glioma cells and human glioblastoma cells, followed by a reduction in target protein expression of around 20% [140]. Apart from the aforementioned dendrimers, carbosilane dendrimers have also been applied for in vivo siRNA delivery to the brain. It was shown that carbosilane dendrimers successfully delivered siRNA to HIV-infected human primary astrocytes and obtained efficient gene silencing without inducing cytotoxicity [139], exhibiting a great potential of transporting siRNA into the brain.

Nonviral gene remedy of Parkinson’s disease (PD) is challenging because of the low transfection efficiency of nonviral gene carriers. Thus, lactoferrin (Lf)-modified PEGylated PAMAM (PAMAM-PEG-Lf) was developed for brain gene delivery [119]. Clathrin-dependent endocytosis, caveolae-mediated endocytosis, and macropinocytosis were involved in the assimilation of PAMAM-PEG-Lf NPs by brain capillary endothelial cells (BCECs), indicating that both receptor- and adsorptive-mediated endocytosis mechanisms contribute to their cellular uptake. The intracellular trafficking outcomes revealed that PAMAM-PEG-Lf NPs could promptly enter the acidic endolysosomal compartments within 5 min and then partially get away within 30 min [120]. The widespread expression of an exogenous gene in the brain was observed after intravenous injection. When PAMAM/DNA weight ratio was 10:1, brain gene expression of the PAMAM-PEG-Lf/DNA complex was approximately 2.3 times higher than that of the PAMAM-PEG-Tf/DNA complex [119]. Using human glial cell line-derived neurotrophic factor gene (hGDNF) as the model gene, multiple dosing of PAMAM-PEG-Lf/DNA could significantly upgrade locomotor activity, could curtail dopaminergic neuronal loss, and could enhance monoamine neurotransmitter levels on both rotenone-induced PD rats and 6-hydroxydopamine-lesioned PD rats, demonstrating powerful neuroprotective effects [117,118]. These findings indicated PAMAM-PEG-Lf can be a potential nonviral gene vector for brain gene delivery via noninvasive administration.

### 5.3. Brain Drug Delivery with Dendrimers for Combination Therapy

**Figure 4:**
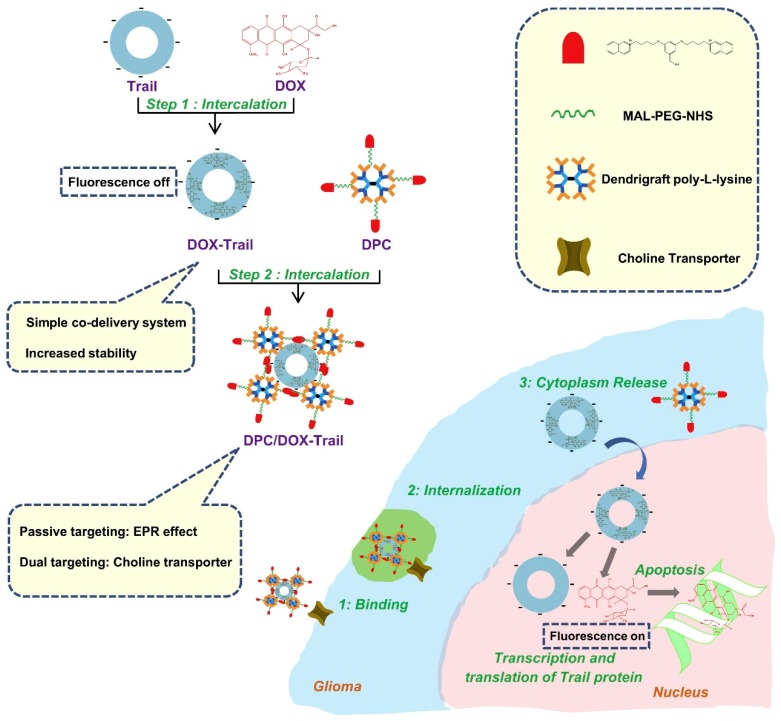
Construction of dual targeting and co-delivery system: Doxorubicin (DOX) was embedded into the TRAIL (tumor necrosis factor-related apoptosis-inducing ligand) plasmid to generate a steady composite, which was further condensed by choline-derivate modified DGL. This co-delivery system could accumulate into glioma cells through EPR and dual targeting effect. After cytoplasm releasing, TRAIL and DOX exert combination therapy on glioma. Reproduced with permission from Reference [134].

Since combination of two or more drugs which have additive or synergistic action may bring about more effective regimens and may prevent the emergence of drug resistance of microorganisms or the tumor, combination therapy has become obligatory in multitude neurological or brain cancer diseases [166]. As for intractable brain tumors, the most common strategies comprise the combination of chemotherapeutic agents to magnify the lethality on tumors and other antitumor agents to alter the tumor microenvironment or to kill tumor cells via multiple pathways [167]. For instance, microRNAs have been shown to be deregulated in disparate types of cancer, in virtue of the fact that downregulation of miR-21 in glioblastoma cells may inhibit cell growth and may increase cell-cycle arrest and cellular apoptosis, which in theory could promote the efficacy of chemotherapy in cancer therapy [168,169]. Ren et al. employed the PAMAM dendrimer as a vehicle to co-deliver antisense-miR-21 oligonucleotide (as-miR-21) and 5-fluorouracil (5-FU) to glioblastoma cells and to enhance the cytotoxicity of 5-FU [168]. Ultimately, the co-delivery of as-miR-21 significantly increased the cytotoxicity of 5-FU and markedly facilitated the apoptosis of U251 cells, while the migration capability of the cancer cells was waned. It has been demonstrated that DOX could regulate the expression of death receptors and improve the antitumor effect of tumor necrosis factor-related apoptosis-inducing ligand (TRAIL) [170]. Thus, the co-delivery of DOX and a therapeutic gene encoding human tumor necrosis factor-related apoptosis-inducing ligand (pORF-hTRAIL) may result in synergistic effect for brain tumor therapy. Li et al. developed a choline transporter-mediated dual targeting co-delivery system of pORF-hTRAIL and DOX for glioma therapy [134]. In their work (Figure 4), DOX was embedded into pORF-hTRAIL, forming a steady composite, which was condensed by choline derivate-modified DGL to construct a nanoparticle co-delivery system. Since choline transporters are expressed on both the glioma and the BBB, the co-delivery system could deliver pORF-hTRAIL and DOX simultaneously to glioma cells after traversing the blood–brain barrier. Distinguished from single medication or unmodified delivery systems, the choline derivate-modified co-delivery system induced more apoptosis in U87 MG cells both in vitro and in vivo, confirming the ascendancy of this dual targeting co-delivery system. Similarly, the co-delivery of pORF-hTRAIL and DOX can also enhance the therapeutic outcome of brain tumors using dual targeting T7 peptide-modified PEGylated PAMAM [122] or DGL [130] dendrimer as the co-delivery platform. Likewise, the efficient co-delivery of pEGFP-hTRAIL gene and paclitaxel (PTX) or actinomycin D to glioma with dendrimers might be another potential drug delivery strategy against glioma [171,172]. Such co-delivery systems may dilate the therapeutic window and allow for the selective destruction of brain tumor cells.

### 5.4. Brain Drug Delivery with Dendrimer-Based Hybrid Nanoparticles

Marked as synthetic, globular, and monodispersed with spatial arrangement and nanometric macromolecules, dendrimers can be further connected to disparate vehicles for various pharmaceutical and biomedical applications [31,173]. To fathom and settle obstacles in drug delivery and other biomedical issues such as side effects, nonnegligible biotoxicity, restricted penetration, and limited drug loading capacity, an increasing number of hybrid carriers based on dendrimers have popped up for brain drug delivery. Recently, Muniswamy et al. synthesized dendrimer-dationized-albumin (D-Alb) following the carboxyl activation technique [174]. The obtained D-Alb was then encrusted on DOX-loaded poly(lactic-co-glycolic acid) (PLGA) nanoparticles (D-Alb@NP-DOX) to generate a neoteric hybrid nanoformulation for brain tumor treatment. It is worth noting that D-Alb@NP-DOX provided excellent antitumor activity of DOX in glioblastoma cells while significantly enhanced its BBB permeability. The dendrimer was also hybridized with quantum dots (QDs, known as the luminescent semiconductor ~9.5 nm) to turn down the cytotoxicity of QDs, improving its water solubility as well as quantum yield [175]. Aiming at efficiently stipulating the accurate dopamine concentration in a customizable manner for assessing Parkinson’s disease, a glass surface was modified on a quantum dot (QD)-encapsulated dendrimer, forming a hybrid biosensor to evaluate the dopamine concentration [176]. Such chemically modified dendrimer-QDs is also good at identifying and tracking neural stem cells as they migrate [177,178]. For the sake of constructing an effective gene delivery system with transmembrane capability for the gene therapy of brain tumors, PAMAM and Tat peptides were conjugated to bacterial magnetic nanoparticles (Tat-BMPs-PAMAM), which were then complexed with small interfering RNA expression plasmid of the human epidermal growth factor receptor gene (psiRNA-EGFR) through electrostatic interplay. The results revealed that Tat-BMPs-PAMAM/psiRNA-EGFR significantly suppressed the expression of oncoproteins and tumor growth compared with control groups, indicating that Tat-BMPs-PAMAM, with its targeted delivery and transmembrane capability, might be a promising gene delivery system with underlying applications in the targeted gene therapy of cerebral tumors [129]. Nanoparticles of large size (100 nm) are characterized with weak tumor penetration but favorable pharmacokinetics, while nanoparticles of small size (<20 nm) result in poor tumor retention but strong tumor penetration. Tumor microenvironment-responsive dendrimer-gelatin hybrid nanoparticles [178] or multistage-responsive hybrid nanoparticles based on dendrimers [179,180] may favor the nanoparticle accumulation in brain tumors, may release small dendrimer nanoparticles to enhance drug penetration in tissue, and may improve brain tumor treatment. Inflammation involves immune cells, blood vessels, and molecular mediators directed against detrimental stimuli, so biomimetic NPs that mimic immune cells could help deliver drugs to these inflammatory sites precisely, such as brain inflammation [181]. Coating dendrimer-based nanoparticles with immune cell membranes may drive future development for improving brain drug delivery [182,183,184]. Hence, combining different components in a single hybrid nanosystem may bring about a novel generation of multifunctional NPs with immaculate structural and biological features. In other words, such NPs not only possess beneficial properties of all the bulk materials but also can be tuned in terms of structural and functional moieties to offer carriers with improved brain-targeting efficiency.

## 6. Concerns and Future Perspectives

As the in-depth research concerning nanobiotechnology continues to spring up, more and more novel inventions of dendrimer-based nanomaterials have significantly influenced and broadened the drug delivery field over the past few decades. However, even if dendrimer-based polymers escort diverse preponderances such as the ability to transport drugs across the BBB or prolong retention time in the circulatory system, their applications in a clinical scenario are restrained in virtue of certain limitations. Besides, as a proof-of-concept, various well-designed dendrimer systems that have been navigated across the BBB are limited only in vitro or in vivo animal models. Hence, there are a handful of concerns that entail to be discreetly addressed for the ultimate translation of dendrimer-based nanomaterials. Notwithstanding the fact that dendrimer-based materials may allow for more effective cellular and subcellular targeting of drugs, unmodified dendrimer-based polymers, especially those with free peripheral amine groups, are extensively reported to evoke concentration- and generation number-dependent cytotoxicity, so their toxicities need attentive monitoring and prudent controlling [31,185,186]. Additionally, comparatively little is known to date on the effects that exposure to dendritic materials may have on the human body, in general, and, specifically, on the brain [187]. Most studies on nanotoxicity do not consider that nanoparticle batches may be contaminated with toxic or bioactive substances, so the reported data on a dendrimer-based system’s toxicity must be criticized. Actually, the long-term safety issues and unforeseeable drug release kinetics associated with dendrimers impose restrictions on their application in brain drug delivery. Interplay between the surface cation charge of dendritic molecules and the negative charge of erythrocyte membrane, which is termed hemolytic toxicity, has also been probed [188]. Several researches have revealed that chemical modifications to remove positively charged amine groups from dendritic polymer surfaces have been shown to mitigate electrostatic cellular interplays and cytotoxicity [188,189]. For example, carboxylation, PEGylation, or acetylation of terminal amine groups on dendritic polymers induces lower cytotoxicity and membrane permeability [190]. Further studies suggest that the toxicity of dendrimers mainly rely on generation, concentration, incubation time, and type of terminal group present on their surface [191,192]. So far, surface modification has been regarded as a sound design tactic to control cell interactions and to alleviate neurotoxicity of dendrimer-based biopolymers.

In addition to its potential neurotoxicity, the fleet systemic clearance of dendritic macromolecules impedes their application in drug delivery. Even trickier, smaller dendritic macromolecules (G2–G4) can be rapidly removed via renal filtration because of their tiny size. On the other hand, larger dendrimers are inclined to be identified and cleared by the reticuloendothelial system [193]. PAMAM and PPI dendrimers exhibited a rapid clearance from blood circulation via the mononuclear phagocyte system, and a high percentage of administrated dendrimers would be accumulated in the liver, kidney, and spleen in terms of dendrimer generation and surface. To figure out these problems, surface functionalization with disparate neutral moieties like PEG chains, antibody, and vitamin on dendrimers will increase their biocompatibility, extend the circulation time [194], and even induce site-specific delivery [192].

Moreover, the interplay of dendrimer-based nanoparticles with the human brain is also an eye-catching nanosafety issue, which is of particular interest. Such interplay may be among the contributing factors in the initiation or progression of neurodegenerative and neuroinflammatory pathologies [195]. Moreover, concurring factors, like individual health and metabolic conditions or the presence of other agents (either physically associated to nanoparticles or co-present during exposure) are additionally modulating the eventual effects of vectors on CNS [196]. In the context of this knotty problem, we may also find that, due to the limited reliability of the available data and paucity of experimental models (in vivo or in vitro in animal cells or human cells) utilized to certify and comprehend the biological effects of dendrimers, we must know that we cannot accurately characterize it at the moment of the experiment in terms of chemical contaminants, ion release, and of major significance, biological contaminants [197].

Another boundedness may be the utilization of dendrimer. Although some biodegradable dendrimers have exhibited significant superiority, most biodegradable dendrimers are polyester dendrimers and a number of them withstand undesired hydrolysis, let alone the degradation of polyesters to fabricate acid by-products, thereby causing local inflammation. Ideally, dendrimers with high biosafety should be degraded into nontoxic compounds that can be absorbed by the biological system or excreted from the body after the completion of therapeutic tasks. Therefore, it is necessary to explore the synthesis and application of new families of biodegradable dendrimers to address such issues.

Overall, the ever-growing development in dendrimer chemistry has streamlined the synthesis of dendrimers, which, in turn, could perfect the scalability and reproducibility of dendrimer-based materials. Although these aforementioned concerns did impede their progress in clinical translation, it is envisaged and expected that some of the highly encouraging dendrimers and dendritic polymers will eventually be translated to be clinically far-reaching [35]. 

## 7. Conclusions

Dendrimers, especially the PAMAM dendrimer invented by Donald A. Tomalia, have garnered extensive attention and paved the path for delivery of agents in spatial-, temporal-, and dosage-controlled fashions for brain targeting. With significant potential as a protean DDS for small molecules, genes, oligonucleotides, proteins, and peptides, dendrimers turn out to be a fascinating nano-vector for the remedy as well as diagnosis of cerebral diseases as a promising nanomedicine incorporating all three elements: functionalization, targeting, and imaging [148]. Although some dendrimer-based systems have not been successful when being clinically translated, several new and hopeful nanoparticles are currently in development and exhibit great promise, thus providing hope for new therapy options in the near future [198]. As for neurological diseases, the noninvasive approach to drug administration as well as enhanced treatment efficacy and diminished side effects would greatly contribute to the preponderance provided by nanosystems toward traversing the BBB. In addition, the usage of neoteric tools for imaging and manipulating the brain will continue to advance our understanding and to provide direct insight into how the human brain gives rise to thought and action; the great potential of dendrimer-based nanocarriers will no doubt boost new dependable means to precisely deliver disparate drugs to the brain.

## Figures and Tables

**Table 1:** An overview of disparate dendrimers involved in targeting drug delivery to brain diseases.

Dendrimers	Ligands	Targeting Pattern	Drug	Diseases	References
G5 PAMAM	Folic acid	Dual-functional glioma targeting	Borneol	Glioma	[116]
G5 PAMAM	Lactoferrin	Targeting the BBB	Plasmid hGDNF	PD	[117,118]
G5 PAMAM	Lactoferrin	Targeting the BBB	pEGFP-N2, pGL2	-	[119,120]
G5 PAMAM	Transferrin	Targeting the BBB	pEGFP-N2, pGL2	-	[121]
G5 PAMAM	HAIYPRH peptide	Targeting the BBB and tumor cells	pORF-hTRAIL, doxorubicin	Glioma	[122]
G4 PAMAM	Transferrin, Tamoxifen	Dual targeting to the BBB and tumor cells	Doxorubicin	Glioma	[123]
G5 PAMAM	Angiopep-2 peptide	Dual targeting to the BBB and tumor cells	pORF-hTRAIL, pGL2	Glioma	[124]
G5 PAMAM	Angiopep-2 peptide	Targeting to the BBB	pEGFP-N2	-	[125]
G5 PAMAM	RVG29 peptide	Targeting to the BBB	pEGFP-N2, pGL2	-	[126]
G5 PAMAM	Chlorotoxin	Targeting to tumor cells	pORF-hTRAIL	Glioma	[127]
G4 PAMAM	SRL peptide	Targeting to the BBB	pEGFP-N2	-	[128]
G4 PAMAM derivate	-	-	HMGB1 siRNA	Cerebral ischemia	[91]
G3 PPI	Transferrin	Targeting to the BBB	pGL	Glioma	[114]
G5 PPI	Sialic acid	Targeting to the BBB	Paclitaxel	-	[112]
G5 PPI	Polysorbate 80	Targeting to the BBB	Docetaxel	Glioma	[129]
G3 DGL	HAIYPRH (T7) peptide	Dual targeting to the BBB and tumor cells	pORF-hTRAIL, doxorubicin	Glioma	[130]
G3 DGL	HAIYPRH (T7) peptide	Dual targeting to the BBB and tumor cells	siRNA for luciferase	Glioma	[131]
G3 DGL	Angiopep peptide	Targeting to the BBB	Plasmid hGDNF	PD	[132]
G3 DGL	Choline derivate	Targeting to the BBB	pGL3	-	[133]
G3 DGL	Choline derivate	Dual targeting to the BBB and tumor cells	pORF-hTRAIL and doxorubicin	Glioma	[134]
G3 DGL	NL4 peptide, apolipoprotein A-I	Dual targeting to the BBB and neurons	BACE1 siRNA	AD	[135]
G3 DGL	Transferrin, MAN	Dual targeting to the BBB and tumor cells	Doxorubicin	Glioma	[136]
PEI-PLL	Angiopep-2	Dual targeting to the BBB and tumor cells	HSV-TK plasmid	Glioma	[137]
PEI-PLL	-	Targeting to dopaminergic neurons	VEGF plasmid	PD	[138]
Carbosilane dendrimer	-	Targeting to the primary astrocytes	siRNA against HIV-1 Nef	HIV	[139]
Amino-functional polyester dendrimers	-	Targeting to the BBB	siRNA	Astrocytes, glioma cells	[140]

PAMAM, polyamidoamine; PPI, polypropyleneimine; hGDNF, human glial-derived neurotrophic factor gene; PD, Parkinson’s disease; EGFP, enhanced green fluorescent protein; HAIYPRH (T7), a TfR-targeting peptide, having a high affinity for the TfR with a Kd of ~10 nM; pORF-hTRAIL, a therapeutic gene encoding human tumor necrosis factor-related apoptosis-inducing ligand; pGL, a report gene encoding wildtype firefly luciferase; HMGB1, high mobility group box-1; NL4, a peptide which can bind with tyrosine kinases A; RVG29, a 29-amonic acid peptide derived from rabies virus glycoprotein; DGL, dendri-grafted poly-l-lysine; SRL, Serine–Arginine–Leucine; BACE1, beta-amyloid converting enzyme 1; AD, Alzheimer’s disease; HSV-TK, herpes simplex virus type I thymidine kinase gene; VEGF, vascular endothelial growth factor; MAN, P-aminophenyl-α-d-mannopyranoside.
